# A universal symmetry detection algorithm

**DOI:** 10.1186/s40064-015-1156-7

**Published:** 2015-08-14

**Authors:** Peter M Maurer

**Affiliations:** Department of Computer Science, Baylor University, Waco, TX 76798-7356 USA

**Keywords:** Symmetry detection, Symmetric Boolean functions, Generalized symmetry

## Abstract

Research on symmetry detection focuses on identifying and detecting new types of symmetry. The paper presents an algorithm that is capable of detecting any type of permutation-based symmetry, including many types for which there are no existing algorithms. General symmetry detection is library-based, but symmetries that can be parameterized, (i.e. total, partial, rotational, and dihedral symmetry), can be detected without using libraries. In many cases it is faster than existing techniques. Furthermore, it is simpler than most existing techniques, and can easily be incorporated into existing software. The algorithm can also be used with virtually any type of matrix-based symmetry, including conjugate symmetry.

## Background

A symmetric Boolean function is a function whose inputs can be rearranged in some fashion without changing the output of the function. The importance such functions was first recognized by Shannon in (Shannon 1949), who characterized function symmetries using permutations of the input variables. Since that time, the detection and exploitation of symmetric Boolean functions has been of recurring interest in the field of design automation (Abdollahi 2006; Biswas 1970; Born and Scidmore 1968; Butler et al. 2000; Chrzanowska-Jeske 2001; Chung and Liu 1998; Darga et al. 2008; Drechsler and Becker 1995; Hu and Marek-Sadowska 2001; Hu et al. 2008; Ke and Menon 1995; Kettle and King 2008; Kravets and Sakallah 2002; Maurer 2011; Mohnke et al. 2002; Moller et al. 1993; Muzio et al. 2008; Rice and Muzio 2002; Scholl et al. 1997; Tsai and Marek-Sadowska 1996; Wang and Chen 2004; Zhang et al. 2004). Virtually all of these algorithms are based on Shannon’s Theorem (Shannon 1949) which detects symmetry by comparison of two-variable cofactors. (See below.) Although comparison of two-variable cofactors is powerful enough to detect all total and partial symmetries, there are many types of symmetries that cannot be detected in this manner. As the number of input variables grows, these types of symmetry become more common than partial and total symmetry. Some progress has been made in detecting symmetries beyond partial and total symmetry (Chrzanowska-Jeske 2001; Tsai and Marek-Sadowska 1994; Kravets and Sakallah 2000), but the problem of universal symmetry detection has remained open since 1949.

**Fig. 1:**
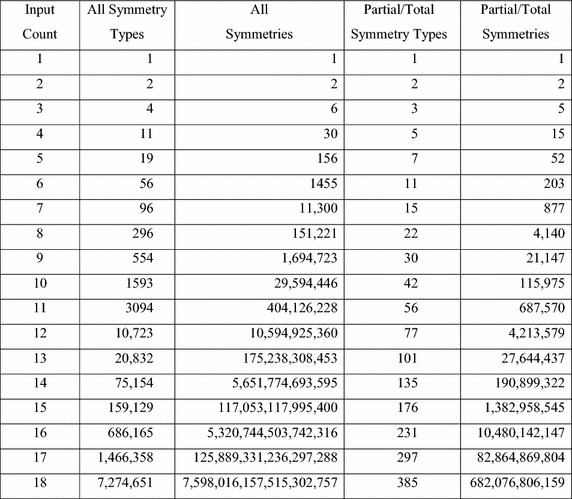
Symmetries and symmetry types (Holt 2010).

**Fig. 2:**
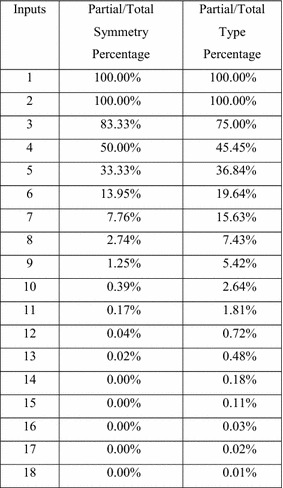
Percentage of partial/total symmetries.

Figure 1 lists the number of symmetries and the number of symmetry types for each number of inputs from 1 through 18. (Two symmetries are of the same type if they are the same but applied to different inputs.) Columns 2 and 3 are the total number of symmetry types and symmetries for each *n*. Columns 4 and 5 are the total number of symmetry types and symmetries for partial and total symmetry. Figure 2 gives the percentage of partial and total symmetries and symmetry types, compared to the total number of symmetries. The data from these tables shows that partial and total symmetries account for less than half of the available symmetry types when the number of inputs is more than 3, and only a tiny percentage of the available symmetries when the number of inputs is 8 or more.

The experiments with standard benchmarks (Brglez et al. 1985) show that symmetries other than partial and total symmetries are common. Other researchers have noted the existence of such symmetries (Kravets and Sakallah 2002; Mohnke et al. 2002). Ignoring such symmetries can cause major failures in layout verification and regression (Maurer and Schapira 1988). These algorithms typically use graph-isomorphism algorithms, starting from the known elements of the circuit (the primary inputs, for example). If a failure occurs early in the process, such as not recognizing that the function $x_{1} x^{\prime}_{2} + x_{3} x^{\prime}_{4}$ in one circuit is identical to the function $x_{3} x^{\prime}_{4} + x_{1} x^{\prime}_{2}$ in the other, the failure can cause a cascade of false errors throughout a major portion of the circuit. When too many false errors are reported, the real errors are extremely difficult to identify. (The function $x_{1} x^{\prime}_{2} + x_{3} x^{\prime}_{4}$ is neither totally nor partially symmetric, but it is obvious the set of inputs $\{ x_{1} ,x_{2} \}$ can be exchanged with the set $\{ x_{3} ,x_{4} \}$ without altering the output.)

Correct handling of symmetry is also important when attempting to match design specifications to an existing library of functions (Mohnke et al. 2002). Suppose the library contains a pre-laid-out implementation of the function $x_{1} x^{\prime}_{2} + x_{3} x^{\prime}_{4}$, and the circuit being laid out contains the subcircuit $x_{3} x^{\prime}_{4} + x_{1} x^{\prime}_{2}$. If symmetry-detection fails, these two functions will not be matched correctly, requiring a new layout (by hand or by machine) for $x_{3} x^{\prime}_{4} + x_{1} x^{\prime}_{2}$, when none is actually required. This can be costly, both in terms of time and of correctness. New implementations must be verified and tested, whereas library implementations are already verified and are much more likely to be correct.

This paper presents an entirely new approach which, effectively, considers all inputs simultaneously instead of in pairs. This approach allows the algorithm to detect virtually any type of symmetry, including some types that go beyond permutations. (These types include matrix-based symmetry, auto-symmetry, Kronecker symmetry, anti-symmetry, and multi-phase symmetry.) For small numbers of inputs (less than 8) the USD is faster than using cofactors. In addition, the coding is simpler. Pseudo code is presented in "The symmetry detection algorithm", which can easily be adapted for use in existing EDA algorithms. The USD algorithm also is somewhat easier to parallelize than the conventional algorithm, because it does not require the accumulation of results to completely characterize a function. The conventional algorithm checks for symmetric variable pairs and then combines the results from these tests to determine the symmetries of a function. Even though each of the symmetric variable pair tests could be done in parallel, combining the results requires some sort of binary fanin or pointer jumping, giving a parallel time bound of O(lg n). The USD tests an entire permutation group in one shot without needing to combine results. Each of these tests could be done in parallel, giving a parallel time bound of O(c).

Despite the advantages of the new approach, the algorithm includes the conventional symmetric-variable-pair detection algorithms as a subset of the new detection algorithms. When no libraries exist for a particular number of inputs, this makes it possible to detect any partial, total, multi-phase, anti, and Kronecker symmetry using the conventional approach.

Symmetries can be categorized into total symmetry, partial symmetry, and strong symmetry. Total symmetry permits the inputs of a function to be rearranged arbitrarily without changing the output of the function. Partial symmetry is similar to total symmetry in that it permits one or more subsets of inputs to be rearranged arbitrarily. Strong symmetry is a catch-all term that includes every type of symmetry that is neither total nor partial. The function $x_{1} + x_{2} + x_{3} + x_{4}$ is totally symmetric, the function $x_{1} x_{2} x_{3} + x_{4}$ is partially symmetric, while the functions $x_{1}^{\prime } x_{2} + x_{3}^{\prime } x_{4}$ and $x_{1} x_{2} + x_{3} x_{4}$ are strongly symmetric. (Functions are specified as expressions in which multiplication signifies AND, addition signifies OR, and the prime symbol specifies NOT.) In $x_{1}^{\prime } x_{2} + x_{3}^{\prime } x_{4}$ no single variable can be exchanged with any other single variable, but the set $\{ x_{1} ,x_{2} \}$ can be exchanged with the set $\{ x_{3} ,x_{4} \}$. The function $x_{1} x_{2} + x_{3} x_{4}$ is more problematical because most existing algorithms will detect two partial symmetries, but ignore the fact that the set $\{ x_{1} ,x_{2} \}$ can be exchanged with the set $\{ x_{3} ,x_{4} \}$. (The algorithm of (Kravets and Sakallah 2002) will detect the correct symmetry for this function.)

There are many more kinds of strong symmetry than partial and total symmetry (See Figs. 1, 2). Various sub-categories of strong of symmetry have been discovered, and algorithms have been created to detect and exploit some of these symmetries (Mohnke et al. 2002). Examples of such symmetries are hierarchical symmetry, rotational symmetry and dihedral symmetry (Kravets and Sakallah 2002).

The primary tool for categorizing symmetry (in any field) is the permutation group (Passman 1968). Let $X$ be a finite set of objects. A permutation is a one-to-one function from $X$ to itself. In other words, a permutation rearranges the elements of $X$ without creating or destroying any elements. Permutations can be “multiplied” using function composition. If $p$ and $q$ are permutations, then so is $pq$ where $(pq)(x) = q(p(x))$. The multiplication operation is associative, ($p(qr) = (pq)r$), but not necessarily commutative, ($pq$ need not equal $qp$). A set of permutations, $G$, that is closed under multiplication (for all $a,b \in G$, $ab \in G$) is called a *Group*. The set of all permutations of a set $X$ is called the *symmetric group* on $X$ and is written $S_{X}$.

Although it is possible to apply permutations to any finite set, the only thing that affects the structure of $$ S_{X} $$ is the size of $$ X $$. If $$ X $$ and $$ Y $$ are two sets of the same size, then $$ S_{X} $$ and $$ S_{Y} $$ are identical. If $$ p \in S_{X} $$, and the size of $$ X $$ is $$ n $$ then $$ n $$ is the *degree* of $$ p $$. If $$ X = \{ 1,2, \ldots ,n\} $$$$ S_{X} $$ is written as $$ S_{n} $$. When speaking of the input variables of a function, the variables will be designated as $$ x_{1} ,x_{2} , \ldots ,x_{n} $$. All permutations are assumed to be elements of $$ S_{n} $$, and permute the variables $$ x_{1} ,x_{2} , \ldots ,x_{n} $$ by operating on their indices.

Every permutation group $G$ has two important properties which are implied by $G$ being closed under multiplication. First, the identity permutation, $I$, is a member of every group. ($Ip = pI = p$ for all $p$.) Second, every permutation $p \in G$ has an *inverse* permutation $p^{ - 1} \in G$ such that $pp^{ - 1} = p^{ - 1} p = I$.

Permutations can be specified in many ways, but in this paper cycle notation will normally be used. Every permutation in $S_{n}$ can be characterized as one or more cyclic shifts of some subset of the integers {1, 2, …, n}. For example the permutation $(1,2,3) \in S_{3}$ maps 1 to 2, 2 to 3, and 3 to 1. A permutation may perform several cyclic shifts simultaneously, as in $(1,2,3)(4,5) \in S_{5}$, which shifts 1, 2, and 3 cyclically and also swaps 4 and 5. Elements that are not moved by the permutation are normally omitted from the cycle notation, so the permutation $(1,2,3)(4)(5)(6) \in S_{6}$ would normally be written (1, 2, 3). In a cyclic shift, it doesn’t matter which element comes first, so the cycles (1, 2, 3), (2, 3, 1), and (3, 1, 2) all denote the same permutation. To avoid this ambiguity, the smallest element of a cycle is always listed first. A similar ambiguity occurs when a permutation performs two or more cyclic shifts of the same size, as in (1, 2, 3)(4, 5, 6). In this case the cycles of the same length are written in ascending order by their smallest elements.

Cycles of length two, such as (1, 2) and (2, 3) are called *transpositions*. Any cycle of length $k$ can be factored into $k - 1$ transpositions in the following manner: $(c_{1} ,c_{2} ,c_{3} \ldots ,c_{k} ) = (c_{1} ,c_{2} )(c_{1} ,c_{3} ) \ldots (c_{1} ,c_{k} )$. This implies that any permutation can be factored into a product of transpositions. This factorization is not unique, but if a permutation, *p,* can be factored into an even number of transpositions, then there is no way to factor it into an odd number of transpositions. By the same token if a permutation can be factored into an odd number of transpositions, then there is no way to factor it into an even number of transpositions. Thus it is possible to characterize each permutation as either even or odd. The product of two odd or two even permutations is even and the product of an odd permutation with an even permutation is odd. For every symmetric group $S_{n}$, there is a subgroup $A_{n}$, consisting of the even permutations of $S_{n}$. The group $A_{n}$ is called the *alternating group* of degree *n*.

Let *p* be a permutation of degree *n* and let *f* be an *n*-input Boolean function. The permutation *p* and the function *f* are *compatible* if using *p* to rearrange the variables of $f$ leaves the output of *f* unchanged. The function *f* is also said to be *invariant with respect to p.* This terminology is extended in the obvious way to subgroups of $S_{n}$. The *symmetry group*$G_{f}$ is the set of *all* permutations that leave *f* invariant. The group $G_{f}$ is closed under multiplication because if $p$ and $q$ leave $f$ invariant, then so does $pq$. Thus $G_{f}$ is a subgroup of $S_{n}$. Because the identity element leaves every function invariant, $G_{f}$ is never empty. Most recognized types of symmetric functions can be characterized using symmetry groups. For example, an *n*-input function $f$ is totally symmetric, if and only if $G_{f} = S_{n}$. A function is *non*-*symmetric* if and only if $G_{f} = \{ I\}$.

Most existing symmetry-detection algorithms use symmetric variable pairs, which are detected by comparing the cofactors of a function (Chrzanowska-Jeske 2001). A cofactor of $f$ is found by setting one or more input variables to constant values. For example, let $f = x_{1} x_{2} + x_{3} x_{4}$. Two cofactors of $f$ are $f_{xx1x} = x_{1} x_{2} + x_{4}$ and $f_{0xxx} = x_{3} x_{4}$. The four positions in the subscript correspond to the four input variables $x_{1}$, $x_{2}$, $x_{3}$, and $x_{4}$ respectively. The subscript indicates which variables have been set to constants and which are unaffected. When the unaffected variables are obvious, it is common to omit the x’s.

Symmetric variable pairs are pairs of variables that can be exchanged without affecting the output of the function. Shannon’s theorem (Shannon 1949) states that $(x_{1} ,x_{2} )$ is a symmetric variable pair if and only if $f_{01} = f_{10}$. Symmetric variable pairs are transitive, which means that if $(x_{i} ,x_{j} )$ and $(x_{j} ,x_{k} )$ are symmetric variable pairs, then so is $(x_{i} ,x_{k} )$. Because of this, all partial and total symmetries can be detected using symmetric variable pairs. However, symmetric variable pairs cannot be used to detect strong symmetries.

This paper introduces a new method of categorizing permutation groups called *Boolean Orbits*. Boolean orbits are used as the basis of a new symmetry detection algorithm that can determine a function’s compatibility with any permutation group. The concept of Boolean orbits can be extended to virtually all types of known symmetry, allowing these types of symmetry to be detected by the algorithm described here.

## Results and discussion

### Boolean orbits

Orbits have been used by mathematicians for many years to analyze and categorize permutation groups (Mohnke et al. 2002; Passman 1968). They have also been used to some extent to analyze symmetric Boolean functions (Mohnke et al. 2002). Orbits are computed as follows. Let *G* be a permutation group that is compatible with a Boolean function $f$, and let $X$ be the set of input variables of $f$. Two variables $x_{i} ,x_{j} \in X$ are said to be in the same *orbit of G* if there is a permutation $p \in G$, such that $p(x_{i} ) = x_{j}$. Intuitively, an orbit contains all the variables that can be exchanged with one another, so the function $x_{1} x_{2} x_{3} + x_{4}$ has two orbits $\{ x_{1} ,x_{2} ,x_{3} \}$ and $\{ x_{4} \}$. Belonging to the same orbit is an equivalence relation, so it breaks the set of input variables into a collection of disjoint subsets.

Orbits can be used to distinguish total and partial symmetries, but are not particularly effective with strong symmetries. Consider the function $$ x_{1} x_{2} + x_{3} x_{4} , $$ which possesses dihedral symmetry. At first it may appear that this function has two orbits, but in fact it has only one, $$ \{ x_{1} ,x_{2} ,x_{3} ,x_{4} \} $$. By the same token, the totally symmetric function $$ x_{1} + x_{2} + x_{3} + x_{4} $$ has a single orbit, $$ \{ x_{1} ,x_{2} ,x_{3} ,x_{4} \} $$. Thus the functions $$ x_{1} + x_{2} + x_{3} + x_{4} $$ and $$ x_{1} x_{2} + x_{3} x_{4} $$ have the same orbits even though their symmetries are quite different.

(Strictly speaking, orbits are properties of permutation groups, not of functions. Thus, the orbit $$ \{ x_{1} ,x_{2} ,x_{3} ,x_{4} \} $$ is the orbit of the *permutation group* of $$ x_{1} x_{2} + x_{3} x_{4} , $$ not of the function itself.)

This paper presents a new type of orbits, called *Boolean Orbits*, that permit one to deal effectively with strong symmetries as well as partial and total symmetries. Boolean orbits are computed with respect to the Boolean input vectors of a function rather than with respect to the variables. (Again, it is important to note that Boolean orbits are properties of *permutation groups*, not of Boolean functions.) Permutations of degree $n$ can operate on *n*-element vectors by permuting the indices of the elements. For example, one can apply the permutation (1, 2, 3) to the vector $(v_{1} ,v_{2} ,v_{3} )$ to obtain $(v_{3} ,v_{1} ,v_{2} )$. Applying this permutation to the specific vector (1, 1, 0) yields the vector (0, 1, 1). The concept of Boolean orbits is formalized in the following definition.

#### **Definition 1**

Given a permutation group *G* of degree *n*, two $n{\text{ - input}}$ vectors $v{\text{ and }}w$ are in the same *Boolean orbit* of $G$ if there is a permutation $p \in G$ such that $p(v) = w$.

**Fig. 3:**
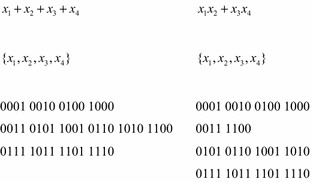
Orbits, and Boolean orbits.

Like ordinary orbits, belonging to the same Boolean Orbit is an equivalence relation, so this relation breaks the set of $n{\text{ - input}}$ Boolean vectors into a collection of disjoint sets. If $G_{f}$ is the symmetry group of a Boolean function, $f$, then the Boolean orbits of $G_{f}$ will partition the truth-table of $f$ into disjoint sets. In fact, the symmetry of a Boolean function is *completely determined* by the Boolean orbits of its permutation group. Figure 3 shows the symmetry groups and the Boolean orbits of the two functions $x_{1} + x_{2} + x_{3} + x_{4}$ and $x_{1} x_{2} + x_{3} x_{4}$. The first two lines of Fig. 3 give the function and the conventional orbits of the function. The final part of Fig. 3 contains the Boolean orbits of the function with one Boolean orbit per line. The Boolean orbits of the two functions are quite different, even though the conventional orbits are the same. (When listing Boolean orbits, all orbits of size 1 are omitted, since these orbits do not affect the symmetry of the function.)

The important properties of Boolean orbits are summarized in the following theorems. Theorem 1 states that a symmetric Boolean function must map the elements of each of the Boolean orbits of its symmetry group to a unique value.

#### **Theorem 1**

*Let*$f$*be a Boolean function, and*$G_{f}$*be the symmetry group of*$f$*. If*$K$*is a Boolean orbit of*$G_{f}$*and*$u,v \in K$*, then*$f(u) = f(v)$.

#### *Proof*

If $K$ is a Boolean orbit of $G_{f}$ and $u,v \in K$, then there is a permutation $p \in G_{f}$ such that $p(u) = v$. Since every element of $G_{f}$ must leave $f$ invariant, $f(u) = f(p(u)) = f(v)$. □

Theorem 2 is the converse of Theorem 1. It states that if the Boolean function,$f$, maps the orbits of a symmetry group, $G$, to unique values, then $f$ is compatible with $G$.

#### **Theorem 2**

*Let*$f$*be an n-input Boolean function and let*$G \subseteq S_{n}$*be a group such that for every Boolean orbit,*$K$*, of*$G$*, and for every pair of elements*$u,v \in K$, $f(u) = f(v)$*then*$f$*is invariant with respect to*$G$*, and*$G \subseteq G_{f}$.

#### *Proof*

Let $p$ be an element of $G$, and let $u$ be any input of $f$. The vectors $u$ and $p(u)$ are in the same Boolean orbit, $K$, of $G$. Since $f(u) = f(v)$ whenever $u,v \in K$, $f(u) = f(p(u))$, and $f$ is invariant with respect to $p$. Since $p$ was arbitrary, every element of $G$ is compatible with $f$. The group $G_{f}$ contains every permutation that leaves $f$ invariant, so if $p \in G$ then $p \in G_{f}$, and $G \subseteq G_{f}$. □

Obviously, the singleton orbits (those containing a single vector) do not affect the symmetry of a function, so when testing a function $f$ for compatibility with a group $G$, the singleton orbits can be ignored.

Theorem 3 deals with the problem of functions that have more than one type of symmetry. As this theorem shows, if a Boolean function $f$ has two different types of symmetry A, and B, then $f$ also possesses an overarching symmetry that includes both A and B. Thus if one can identify the largest symmetry group that is compatible with $f$, then all of the symmetries possessed by $f$ have been discovered.

#### **Theorem 3**

*Let*$f$*be a Boolean function and let*$G$*and*$H$*be two permutation groups that are compatible with*$f$*. Then there is a permutation group, *$K$*compatible with*$f$*such that*$G$*and*$H$*are both subgroups of*$K$.

#### *Proof*

Let $K$ be the smallest subgroup of $S_{n}$ containing $G \cup H$. Since $S_{n}$ contains both $G$ and $H$, $K$ must exist. From group theory it is known that that every element of $p \in K$ is of the form $p = q_{1} q_{2} \ldots q_{k}$ where $q_{i} \in G$ or $q_{i} \in H$. Since every element of either $G$ or $H$ is compatible with $f$, $p$ must also be compatible with $f$, and $K$ is the required group. □

**Fig. 4:**
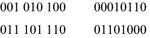
Boolean orbits and characteristic functions.

The remainder of the paper will make extensive use of the *characteristic function* of an orbit. Let $S$ be any set of $n{\text{-element}}$ Boolean vectors. The characteristic function of $S$, $C_{s}$ is an *n*-input Boolean function which is equal to 1 on every element of $S$, and zero elsewhere. Figure 4 gives a set of orbits along with their characteristic functions in truth-table form.

The characteristic functions of the Boolean Orbits can be used to determine the symmetries of a Boolean function.

**Fig. 5:**
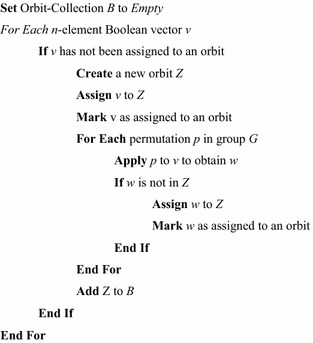
Generating Boolean orbits.

Given a permutation group, *G*, computing the Boolean orbits of *G* is straightforward. The algorithm is given in Fig. 5.

Shannon’s theorem, which is the basis of virtually all other symmetry detection algorithms, is a special case of Theorem 2. Shannon’s theorem deals with symmetric variable pairs of a function $f$, $(x_{i} ,x_{j} )$. Formally, symmetric variable pairs are defined as follows.

#### **Definition 2**

Let $f$ be an n-input Boolean function and let $x_{i}$ and $x_{j}$ be two input variables of $f$. The pair $(x_{i} ,x_{j} )$ is a symmetric variable pair if and only if $f$ is compatible with the permutation group $\{ I,(i,j)\}$.

The proof of Theorem 4 shows that Shannon’s theorem is a special case of Theorem 2 above. (The truth of this theorem is, of course, well known.)

#### **Theorem 4**

*Let*$f$*be a Boolean function, and let*$f_{00}$, $f_{01}$, $f_{10}$*and*$f_{11}$*be the cofactors of*$f$*with respect to the pair of variables*$(x_{i} ,x_{j} )$*. The pair*$(x_{i} ,x_{j} )$*is a symmetric variable pair of*$f$*, if and only if*$f_{01} = f_{10}$.

#### *Proof*

If $(x_{i} ,x_{j} )$ is a symmetric variable pair of $f$, then $f$ must be invariant with respect to the permutation group $\{ I,(i,j)\}$. The Boolean orbits of this group are either singletons of the form {…0…0…}, or {…1…1…}, or pairs of the form {…0…1…, …1…0…}, where the unspecified parts are identical for each vector. The pairs run through all Boolean combinations of the unspecified parts. The function $f$ must map the two elements of each Boolean orbit of the form {…0…1…,…1…0…} to the same value. If all vectors of the form …0…1… are combined into a single set, the 0, and the 1 are ignored, the result is the truth table of the cofactor $f_{01}$. The truth table of the cofactor $f_{10}$ is obtained in the same manner. Because each element of each Boolean orbit must be mapped to a single value, the truth tables must be the identical, implying that $f_{01} = f_{10}$. For the converse, if $f_{01} = f_{10}$, it is possible to expand each of the cofactors into a truth table. These truth tables must be identical. It is then possible to insert 0 and 1 into the appropriate positions of each truth table to obtain the values of $f$ for each of the inputs of the form …0…1… and …1…0…. Now consider two vectors of the form …0…1… and …1…0… where the unspecified parts are identical. These vectors were obtained from a single entry in a single truth table, therefore they must be mapped to the same value by $f$. Therefore $f$ must be invariant with respect to the permutation group $\{ I,(i,j)\}$, and $(x_{i} ,x_{j} )$ must be a symmetric variable pair. □

### The implication relation

Let $f$ and $g$ be two *n*-input Boolean functions. The function $f$ is said to *imply*$g$ if $g(v) = 1$ whenever $f(v) = 1$. The concept of implication to defines the fundamental relationship on which the USD algorithm is based. This result is given in Theorem 5.

#### **Theorem 5**

*Let*$$ G \subseteq S_{n} $$*be a permutation group, and let*$$ f $$*be an n-input Boolean function. Let*$$ K = \{ K_{1} ,K_{2} , \ldots ,K_{n} \} $$*be the collection of Boolean orbits of G with characteristic functions*$$ \{ C_{1} ,C_{2} , \ldots ,C_{k} \} $$*. The group*$$ G $$*leaves*$$ f $$*invariant if and only if*$$ C_{i} $$*implies either*$$ f $$*or*$$ \overset{\lower0.5em\hbox{$\smash{\scriptscriptstyle\rightharpoonup}$}} {f} $$*for every*$$ i $$, $$ 1 \le i \le k $$.

#### *Proof*

Suppose $C_{i}$ implies $f$, and $v \in K_{i}$. Then $C_{i} (v) = 1$, and because $C_{i}$ implies $f$, $f(v) = 1$ for all $v \in K_{i}$. Now suppose $C_{i}$ implies $\overline{f}$. If $v \in K_{i}$ then $C_{i} (v) = 1$ and since $C_{i}$ implies $\overline{f}$, $\overline{f}(v) = 1$, implying that $f(v) = 0$ for all $v \in K_{i}$. By Theorem 2, $f$ must be invariant with respect to $G$.

Now suppose $f$ is invariant with respect to $G$. By Theorem 1, if $u,v \in K_{i}$ then $f(u) = f(v)$. But $C_{i} (u) = C_{i} (v) = 1$. If $f(u) = f(v) = 1$, then $C_{i}$ implies $f$. If $f(u) = f(v) = 0$, then $C_{i}$ implies $\overline{f}$. □

Theorem 5 gives a principle that can be used to detect symmetry with respect to *any* permutation group. Given a permutation group $$ G $$ it is straightforward to compute the characteristic functions of its Boolean orbits. Given $$ f $$ it is straightforward to compute $$ \overset{\lower0.5em\hbox{$\smash{\scriptscriptstyle\rightharpoonup}$}} {f} $$. Once these functions have been computed, one only need check the characteristic function of each orbit to determine whether $$ f $$ is symmetric with respect to $$ G $$.

Although symmetry detection is normally done on single-output functions, the principle can be extended easily to multiple-output functions. First it is necessary to define the function orbits of multiple output functions.

#### **Definition 3**

Let *f* be a Boolean function with *n* inputs and *m* outputs. For each *m*-element vector *v*, $S_{f}^{[v]}$ is the set of all *n*-element input vectors on which *f* takes the value *v*. The sets $S_{f}^{[v]}$ are known as the *function orbits* of *f*. The function $f^{[v]}$ is the characteristic function of $S_{f}^{[v]}$.

Theorem 6 extends the implication principle to Boolean functions with multiple outputs.

#### **Theorem 6**

*Let*$G \subseteq S_{n}$*be a permutation group, and let*$f$*be an n-input Boolean function with m outputs. Let*$K = \{ K_{1} ,K_{2} , \ldots ,K_{n} \}$*be the collection of Boolean orbits of*$G$*with characteristic functions*$\{ C_{1} ,C_{2} , \ldots ,C_{k} \}$*. The group*$G$*leaves*$f$*invariant if and only if for every*$i$, $1 \le i \le k$, $C_{i}$*implies*$f^{[v]}$*for some m-element vector v.*

The proof is essentially identical to that of Theorem 5.

### The symmetry detection algorithm

**Fig. 6:**
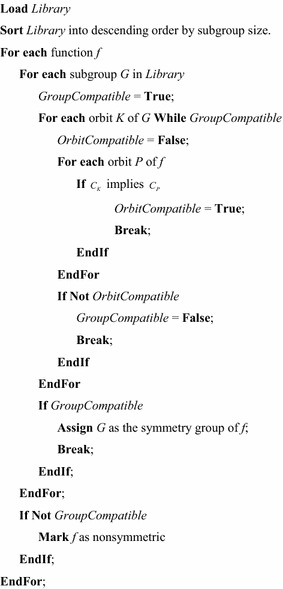
The universal symmetry detection algorithm.

Figure 6 gives the pseudo code for the universal symmetry detection (USD) algorithm. In this figure, it is assumed that the algorithm is being applied to a collection of functions, and that a library of symmetries is being used. Each library entry contains a set of characteristic functions that correspond to the Boolean orbits of a symmetry group. In most cases, the library will contain all subgroups of $S_{n}$ for some integer, $n$, although there are a number of other more specialized libraries. Complete libraries for $S_{2}$ through $S_{8}$ have been created. Subgroup libraries for $S_{9}$ through $S_{18}$ exist on the web (Holt 2010; Pfeiffer 2005), but these have not yet been adapted for use with the USD. When used with a complete library for $S_{n}$ symmetry detection begins with the largest group so the algorithm may stop as soon as a compatible group is found.

As Fig. 6 shows, the algorithm reads each function, and compares it to each library entry until a compatible entry is found. Most libraries contain a “non-symmetric” entry which permits each function to be associated with at least one library entry. However, such an entry is not required. If no compatible subgroup can be found, the function is marked as non-symmetric.

Comparison between a function and a subgroup is done by enumerating the Boolean orbits of the subgroup. Each Boolean orbit is tested against $f$ and $\overline{f}$ seeking an implication. If a particular subgroup orbit does not imply either function orbit, the comparison with the group is terminated. However, if all subgroup orbits imply a function orbit, the subgroup is assigned to the function as its symmetry group, and testing of the function terminates.

Libraries are not necessary for symmetries that can be parameterized for an arbitrary number of inputs. As yet, only a few symmetries have been so categorized, the most well-known of which are total, symmetry, partial symmetry, rotational symmetry, dihedral symmetry, and various types of hierarchical symmetry. The USD algorithm has special generators for total, partial, dihedral and rotational symmetry, which permits these types of symmetries to be detected without having a precomputed library. Of course it is possible to cache the output of these generators for future use.

Most of the existing libraries contain one entry per symmetry group. However, for large numbers of inputs it is not feasible to store libraries in this fashion. (See Fig. 1.) The *conjugacy* relation can be used to reduce the size of the library for large numbers of inputs. Certain types of symmetry are fundamentally the same, but applied to different inputs, and certain types of symmetry are fundamentally different. For example, a 3-input partial symmetry on the first three inputs of a function is not fundamentally different from a three-input partial symmetry on the last three variables. But a partial symmetry in the first three variables *is* fundamentally different from a partial symmetry in the first *two* variables. The conjugacy relation is used to distinguish symmetries that are essentially the same from symmetries that are fundamentally different. Definition 4 permits the formalization this idea.

#### **Definition 4**

Two permutations *p* and *q* are *conjugate* to one another if there is another permutation *s* such that $p = s^{ - 1} qs$.

Conjugacy can be best understood by visualizing it in this way: to permute the last three variables of a function, move them to the first three variables using $s$, then apply $q$ to the first three variables, and then use $s^{ - 1}$ to move the variables back where they were.

This relationship can be extended to permutation groups in the following way: $s^{ - 1} Gs = \{ s^{ - 1} ps|p \in G\} .$ If two symmetries are fundamentally the same then their permutation groups will be conjugate to one another. For example, all partial symmetries on three inputs have conjugate symmetry groups.

Conjugacy is an equivalence relation, so the subgroups of a group can be partitioned into a set of conjugacy classes. The Column 2 of Fig. 1 shows the number of conjugacy classes and Column 3 shows the number of subgroups for the symmetric groups from $S_{1}$ through $S_{18}$. The full libraries store each subgroup of the symmetric group. Reduced libraries store only one member of each conjugacy class.

To regenerate a conjugacy class, it is necessary to compute the conjugates of each library entry. However for each subgroup $G$ of $S_{n}$ there are many pairs of permutations $(p,q)$ such that $p \ne q$, but $p^{ - 1} Gp = q^{ - 1} Gq$. To avoid duplicated work a set of permutations is stored with the library entry. There is one permutation for each conjugate, so applying each permutation to the entry will restore the entire class.

The permutations are computed when creating the library. A group theoretic result states that “the number of conjugates of a group is equal to the index (i.e. number of right cosets) of its normalizer (Robinson 1995).” This is a simple idea, but requires some background discussion.

The *normalizer* of a group, *G,* written $N(G)$, is the set of permutations that leave *G* unchanged with respect to conjugacy. That is, the set $N(G) = \{ p|p^{ - 1} Gp = G\} .$ ($N(G)$ is always a subgroup of $S_{n}$.) Since *G* is closed under multiplication, every element of *G* leaves *G* invariant under conjugation, and *G* is a subgroup of $N(G)$. However, $N(G)$ often contains other elements as well. If $p \notin N(G)$, then $p^{ - 1} Gp \ne G$, but for every element $q \in N(G)$, $(qp)^{ - 1} Gqp = p^{ - 1} q^{ - 1} Gqp = p^{ - 1} Gp$. So, if it is desired to include only a minimal set of permutations in the library entry, it is necessary to avoid including both $p$ and $qp$. In fact, a theorem of group theory states that if two permutations $r$ and $s$ produce the same conjugate of *G,* (i.e. $r^{ - 1} Gr = s^{ - 1} Gs$), then there must be some $q \in N(G)$ such that $qr = s$. This means that the entire set of permutations that produce the same conjugate as permutation $r$ is the set $N(G)r = \{ qr|q \in N(G)\}$. The set $N(G)r$ is called a *right coset* of $N(G)$. Another theorem of group theory states that given two right cosets of $N(G)$, $N(G)r$ and $N(G)s$, either $N(G)r = N(G)s$ or $N(G)r \cap N(G)s = \phi$. So if $r$ and $s$ belong to the same right coset of $N(G)$, then $r^{ - 1} Gr = s^{ - 1} Gs$. If $r$ and $s$ belong to different right cosets of $N(G)$, then $r^{ - 1} Gr \ne s^{ - 1} Gs$. This means that if one can generate all of the right cosets of G, and then select one permutation from each, one will have a minimal set of permutations. Such a set is called a set of *coset representatives.*

In summary:

1. There is set of permutations that don’t change G under conjugation.
2. This set can be used to generate a collection of sets, each one of which contains permutations that all produce the same conjugate of G.
3. Choosing one representative from each of the sets will give a minimal set of permutations.

**Fig. 7:**
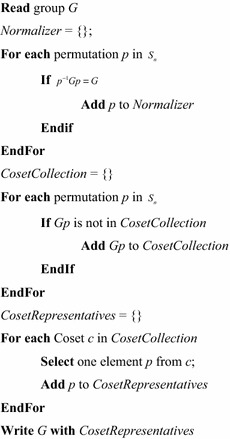
Generating coset representatives.

**Fig. 8:**
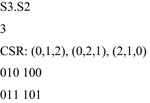
A reduced library entry.

Figure 7 gives the algorithm for creating a reduced library entry. Figure 8 gives the library entry that is used to detect 2-variable partial symmetry in 3-input functions. The permutations are coded in the form of a list of numbers from the set {0, 1, 2}. The first permutation is the identity, *I*.

The first line of Fig. 8 is the name of the symmetry, the second is the number of inputs, the third is the list of coset representatives, and the remainder is the Boolean orbits, one orbit per line.

**Fig. 9:**
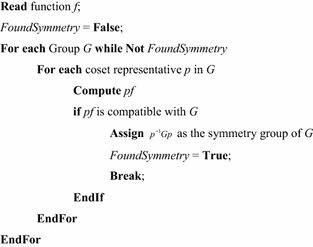
The reduced-library detection algorithm.

For the larger symmetric groups, reconstructing all conjugacy classes is a physical impossibility. So instead, the set of stored permutations is used to alter the function under test. Let’s suppose that $g$ is invariant with respect to $p^{ - 1} Gp$. Then, there is an $f$ which is invariant with respect to $G$ such that $p^{ - 1} f = g$. If $g$ is invariant with respect to $p^{ - 1} Gp$, then $pg$ is invariant with respect to $G$. Figure 9 gives the pseudocode for detecting symmetry with reduced library entries. The test for compatibility in Fig. 9 is identical to that in Fig. 6. In most cases, this code will be slower than using a fully expanded library, so the algorithm of Fig. 9 is used only when necessary.

### Forbidden groups

There are certain permutation groups, and certain matrix groups that cannot be the symmetry group of any function. For example, suppose $G$ is a permutation group with Boolean orbits $B$, and suppose there is another group $H$ such that $G \subset H$($G \ne H$) such that $H$ also has Boolean orbits $B$. In this case, $G$ cannot be the symmetry group of any function, because any function, $f$ that is compatible with $G$ will also be compatible with $H$, but $G$ does not contain every permutation that is compatible with $f$, so $G$ cannot be the symmetry group of $f$.

Groups of this nature are called *forbidden groups*. Forbidden groups can arise in two different ways. Some forbidden groups are *output limited* and some are *input limited.*

More formally, an *output limited* group, *G,* has three or more Boolean orbits of the same weight that must be distinguished from one another to keep a function from being compatible with larger groups containing *G*. However, because the function has only two output values, this is impossible. Output limitations disappear when there are more than two output values.

An *input limited* group, *G,* contains a set of permutations that must be distinguished from those of a larger group containing *G* to keep a function from being compatible with the larger group. However, the permutations of the larger group cannot be so distinguished, because the only thing the larger group adds is a permutations that move the identical elements of each input vector. Input limitations disappear when inputs are allowed to have more than two values.

**Fig. 10:**
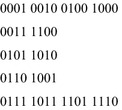
The Boolean orbits of K4.

Input limited groups are easy to detect because they will have the same Boolean orbits as a larger group. Output limited groups are more complicated. For example, the group ${\text{K4}} = \left\{ {{\text{I}}, \, \left( { 1, 2} \right)\left( { 3, 4} \right), \, \left( { 1, 3} \right)\left( { 2, 4} \right), \, \left( {1,4} \right)\left( {2,3} \right)} \right\}$ is output limited. (This group is known as the Klein 4-group, hence the name K4.) The Boolean orbits of K4 are given in Fig. 10.

**Fig. 11:**
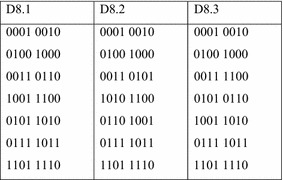
The three conjugates of D8.

These orbits are quite similar to the Boolean orbits of the three conjugates of $D_{8}$, the dihedral group of order 8. These orbits are given in Fig. 11.

**Fig. 12:**
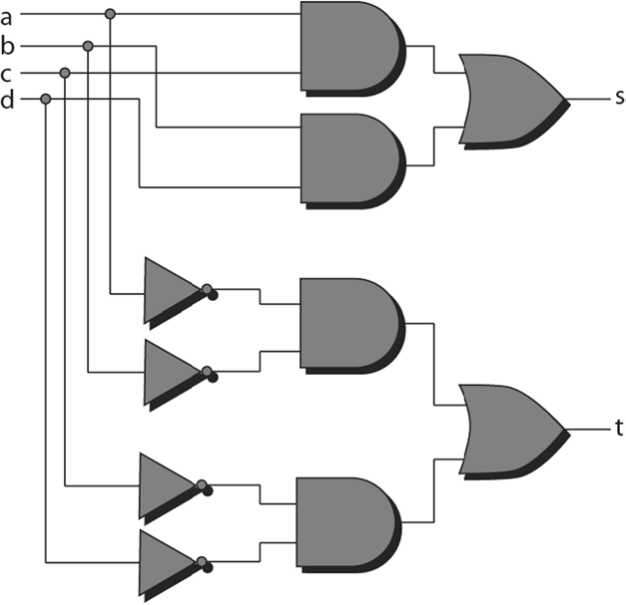
A function with K4 symmetry.

The problem with K4 lies with the three orbits {0011, 1100}, {0101, 1010} and {0110, 1010}. If a function maps the first two orbits to the same value, the result will be dihedral symmetry of type D8.2. If it maps the last two orbits to the same value, the result will be a dihedral symmetry of type D8.3. If it maps the first and last orbits to the same value, the result will be a dihedral symmetry of type D8.1. Because a single-valued function has only two possible output values, at least two of these orbits must be mapped to the same value. Thus no single-valued function can have symmetry K4. On the other hand, it is possible for multiple-valued functions to have K4 symmetry. The function of Fig. 12 is an example. K4 also appears as a sub-symmetry in functions with five or more inputs.

**Fig. 13:**
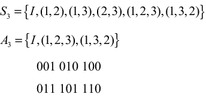
The Boolean orbits of $S_{3}$ and $A_{3}$.

A similar phenomenon occurs with input limited groups. For example, the two symmetry groups $S_{3}$ and $A_{3}$ have identical Boolean orbits, as shown in Fig. 13. Thus $A_{3}$ is forbidden. All of the alternating groups for $n > 2$ are forbidden, as is shown by Theorem 8.

To show that all alternating groups are forbidden, it is necessary to start with the following lemma.

#### **Lemma 1**

*Let n be greater than 2,*$v$*be an n-element vector, and*$p$*be a permutation of degree n. If*$p(v) = w$*, then there is an even permutation*$q$*such that*$q(v) = w$.

#### *Proof*

Suppose $p$ is odd. Because *n* is greater than 2, there must be two elements of $v$ that are identical. Suppose these are positions $i$ and $j$, with $i \ne j$. Thus $(i,j)$ is a 2-cycle, and $q = (i,j)p$ is an even permutation. But $(i,j)(v) = v$, and $q(v) = p((i,j)(v)) = p(v) = w$. □

#### **Theorem 7**

*For all*$$ n > 2 $$$$ A_{n} $$*is forbidden.*

#### *Proof*

Suppose $u$ and $v$ are in the same Boolean orbit of $S_{n}$. Then there must be a $p \in S_{n}$ such that $p(u) = v$. By Lemma 1, there must be a $q \in A_{n}$ such that $q(u) = v$. Therefore $u$ and $v$ must be in the same Boolean orbit of $A_{n}$. Thus every Boolean orbit of $S_{n}$ must be contained in a Boolean orbit of $A_{n}$. Because the Boolean orbits of $S_{n}$ are as large as possible, the Boolean orbits of $S_{n}$ and $A_{n}$ must be identical, and because $A_{n} \subseteq S_{n}$, $A_{n}$ must be forbidden. □

The groups $A_{n}$ are examples of input limited groups. They are forbidden because there are only two distinct values for each input. For functions with multiple-valued inputs, not all of the alternating groups are be forbidden. (Extended versions of Lemma 1 and Theorem 7, would still be true for sufficiently large *n*.)

**Fig. 14:**
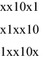
The cubes of $x_{1} x_{4} x_{5}^{\prime } + x_{2} x_{5} x_{6}^{\prime } + x_{3} x_{4}^{\prime } x_{6}$.

As an example, the subgroup of $S_{6}$, $S3a = \left\{ {I,(1,2,3)(4,5,6),(1,3,2)(4,6,5)} \right\}$, is isomorphic to $A_{3}$ (but not conjugate to $A_{3}$). $S3a$ is *not* forbidden, in fact the function $x_{1} x_{4} x_{5}^{\prime } + x_{2} x_{5} x_{6}^{\prime } + x_{3} x_{4}^{\prime } x_{6}$ possesses $S3a$ symmetry. This function is derived from the three cubes of Fig. 14, which make it easy to see that a rotation of the first three variables must be accompanied by a rotation of the last three variables. The reason that $S3a$ is not forbidden is that the inputs operate in pairs: $\{ x_{1} ,x_{4} \}$, $\{ x_{2} ,x_{5} \}$ and $\{ x_{3} ,x_{6} \}$. Each pair of inputs has four possible values, so the argument of Theorem 7 does not apply.

The elimination of forbidden groups from symmetry libraries is an important optimization step, because it eliminates symmetry tests that can never succeed.

Matrix-based symmetry also displays the phenomenon of forbidden groups.

### Sub-symmetries

For functions with many inputs, it may be more useful to detect smaller, more manageable symmetries on a subset of inputs. Such symmetries are called *Sub*-*Symmetries*. The USD algorithm is capable of detecting sub-symmetries using two different techniques.

The first technique is to “promote” existing symmetry rules to a collection of rules for a larger number of inputs. Although this process is technically feasible, it is cumbersome, and can be extremely slow for reduced library entries.

The second procedure alters the functions under test rather than the libraries. It is based on the following Theorem 8.

#### **Theorem 8**

*Let*$R$*be a symmetry group of degree*$k$*, and let*$f$*be a function of*$n > k$*inputs. Let*$S$*be a subset of*$k$*inputs taken from the*$n$*inputs of*$f$*. If*$f$*possesses*$R$*symmetry in the set of*$k$*variables, then every cofactor obtained by fixing the*$n - k$*variables to constant values must possess*$R$*symmetry.*

The Proof is obvious.

**Fig. 15:**
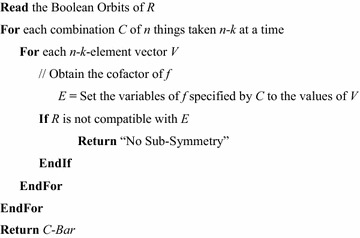
The sub-symmetry detection algorithm.

There are $2^{n - k}$ such cofactors for each set of $k$ inputs. When testing an *n*-input function using a symmetry rule of degree $k < n$, the USD algorithm begins by generating all combinations of $n$ inputs taken $k$ at a time. For each combination, the USD algorithm generates all $2^{n - k}$ cofactors, and tests each one for $R$ symmetry. Figure 15 gives the algorithm for detecting sub-symmetries. If $n - k$ is extremely large, this algorithm may be unacceptably slow, but for small $n - k$, the performance is reasonable.

The algorithm then tests for a sub-symmetry in the $k$ variables selected by the combination. Each such test requires computing $2^{n - k}$ cofactors. These cofactors are computed by setting the variables not selected by the permutation to every possible combination of zeros and ones. The procedure continues until a sub-symmetry is found, or until all combinations have been exhausted. The *C*-*Bar* return value is a combination of *n* things taken *k* at a time that specifies the variables containing the sub-symmetry. It is easily computed from *C*.

The algorithm of Fig. 15 can be easily modified to find multiple sub-symmetries of the same type, however to avoid detecting overlapping symmetries, it is better to run the algorithm of Fig. 15 on cofactors of *f* that do not include the variables specified by *C*-*Bar*.

### Partial symmetries

In some cases, it may be desirable to restrict the focus to partial and total symmetries. These symmetries are well understood, and can be exploited by several existing algorithms. The universal symmetry detection algorithm can be used to detect these types of symmetries either by testing for symmetric variable pairs or by using a library of partial symmetry orbits.

Although partial symmetries are easy to understand, enumerating all of them is a difficult task. For *n* inputs, the number of partial symmetries is equal to the number of partitions of a set of size *n*. A partition of a set *S* is a collection of non-empty sets $P = \{ Q_{1} ,Q_{2} , \ldots ,Q_{k} \}$, such that $Q_{1} \cup Q_{2} \cup \ldots \cup Q_{k} = S$ and $Q_{i} \cap Q_{j} = \phi$ whenever $i \ne j$. The number of partitions of a set of size *n* is given by the Bell number $B_{n}$, where $B_{1} = 1$, and $$ B_{n + 1} = \sum\limits_{i = 0}^{n} {\left( {\begin{array}{*{20}c} n \\ i \\ \end{array} } \right)B_{i} } $$. This formula was used to compute the numbers of partial symmetries listed in Fig. 1.

The number of conjugacy classes of partial symmetries is equal to the number of *integer partitions* of the integer *n*, $p(n)$. Exact formulas for $p(n)$, are known, but they are so complex that, for small *n*, it is easier to generate all integer partitions of *n* and count them. The results for all integers less than or equal to 18 are given in Fig. 1.

To generate a library of all partial symmetries for a specific number of inputs, *n*, it is necessary to generate all partitions of the input variables. Because the Bell numbers provide only a count of the partitions, not the partitions themselves, it is necessary to begin with the integer partitions of *n*. Each integer partition generates a number of partitions. Each partition generates a set of Boolean orbits. The Boolean orbits are stored in a library in function form. When the number of inputs is seven or less, using libraries is faster. For larger numbers of inputs, the USD uses the conventional approach of generating and comparing cofactors to detect symmetric variable pairs.

### Extended symmetric variable pairs

**Fig. 16:**
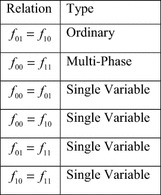
The extended symmetry relations.

Researchers have identified various types of symmetric variable pairs that go beyond those discussed in “Background”. These extended types are defined in terms of the cofactors of a function $f$, with respect to a pair of variables $\{ x_{i} ,x_{j} \}$. As before, these are designated $f_{00}$, $f_{01}$, $f_{10}$, and $f_{11}$. The extended types of symmetry are defined by relations between these four cofactors. Figure 16 lists the six possible relations, and the types of symmetry defined by each.

**Fig. 17:**
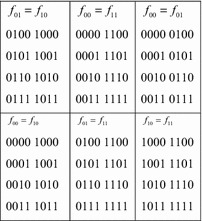
Boolean orbits for extended relations.

It is possible to create Boolean orbits for each of these six relations. Assume that it is desired to detect these symmetries in a four-input function $f$, with respect to the variables $x_{1}$ and $x_{2}$. The Boolean orbits for these six relations are given in Fig. 17.

As pointed out in (Maurer 2011), the single-variable relations are a special case of conjugate symmetry, and are best handled through matrix-based symmetry. Multi-phase symmetry is discussed more thoroughly in the following section.

### Multi-phase symmetry

**Fig. 18:**
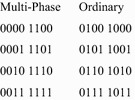
The Boolean orbits of a multi-phase symmetry.

Multi-phase symmetry is the same as ordinary symmetry with one or more inputs inverted with respect to the others. In other words, the inputs of the function are assumed to be a mixture of active-high and active-low inputs. Multi-phase symmetry is normally defined in terms of pairs of symmetric variables, but like ordinary symmetry, it can be defined entirely by Boolean orbits. Suppose it is desired to detect a multi-phase symmetry between the first two variables of a four-input function, $f$. In terms of cofactors, it is necessary to verify that $f_{00} = f_{11}$. This is just the Shannon relation, $f_{10} = f_{01}$, with one of the inputs inverted (it doesn’t matter which one). The Boolean orbits of this relation are given in Fig. 18, along with the Boolean orbits of an ordinary symmetry in the same two variables. (These are the same orbits given in Fig. 17.)

Figure 17 shows that one can obtain the multi-phase Boolean orbits by inverting one of the inputs of the ordinary Boolean orbits. In fact, it is possible invert any subset of the inputs, but not all such inversions will change the orbits. For example, inverting the last two bits of the orbits of Fig. 18 will leave them unchanged.

In terms of vectors, the multi-phase orbits of Fig. 18 were obtained by XORing a constant vector, 0100, to each vector of each orbit as follows. If $S$ is an orbit and $v$ is a vector, then $S \oplus v = \{ w \oplus v|w \in S\}$, where $\oplus$ denotes the XOR operation. This principle can be applied to any set of orbits, not just those representing symmetric variable pairs. Consider the symmetry group $\{ I,(1,2)(3,4)\}$. This type of symmetry cannot be defined in terms of symmetric variable pairs. (The function $x_{1} x_{2}^{\prime } + x_{3} x_{4}^{\prime }$ from “Background” possesses such symmetry.)

**Fig. 19:**
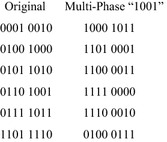
Complex multi-phase symmetry.

It is possible detect multi-phase symmetry in an arbitrary number of variables, by XORing an arbitrary vector with each of the orbits of the original symmetry. Assume that it is desired to detect symmetry of type $\{ I,(1,2)(3,4)\}$ in a function whose first and last variables are active high. To do this, one must add the vector 1001 to all orbits of the original symmetry. Figure 19 shows how this is done.

Computation of multi-phase orbits can be performed on the fly or loaded from a library.

If $v \oplus w = 11 \ldots 1$, the vector of all 1’s, then adding $v$ to a set of orbits will produce the same result as adding $w$ to the same orbits. In practice, only those vectors whose first element is zero are used.

Conventional multi-phase symmetries (i.e. those related to partial and total symmetry) can also be detected by using cofactor relations.

### Anti-symmetry

**Fig. 20:**
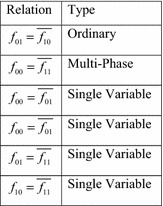
The anti-symmetry relations.

Anti-symmetry (Tsai and Marek-Sadowska 1994) (which is also known as Skew Symmetry and Negative Symmetry) was initially defined in terms of symmetric variable pairs. As with other symmetries, the anti-symmetries are defined with respect to the four cofactors $f_{00}$, $f_{01}$, $f_{10}$ and $f_{11}$, taken with respect to the variables $x_{i}$ and $x_{j}$. The normal symmetric variable pairs are defined by the relations in Fig. 16. The corresponding anti-symmetry relations are given in Fig. 20.

Anti-Symmetry is important, because it is just as common as normal symmetry. For example, an analysis all 4-input functions shows 24,576 examples of ordinary symmetric variable pairs, and the same number of ordinary anti-symmetric variable pairs.

Suppose $f$ has an anti-symmetric variable pair $(x_{i} ,x_{j} )$. If this were an ordinary symmetric variable pair, the symmetry group of $f$ would contain the group $\left\{ {I,(x_{i} ,x_{j} )} \right\}$. To test for this subgroup one would check the orbits {01x…x, 10x…x}, where “x…x” ranges through all bit combinations. However, because this is an anti-symmetric variable pair, $f_{10}$ and $f_{01}$ must produce the opposite value for each input. If 01x…x produces a one value then 10x…x must produce a zero value, and vice versa. Thus, no orbit may imply either $f$ or $f^{\prime}$. This is a necessary and sufficient condition for the anti-symmetric variable pair to exist. Thus, it is possible test for anti-symmetric variable pairs using the same library used to detect normal symmetric variable pairs. When testing the orbits, it is necessary to test for non-implication rather than implication. This is a simple change, enabling the algorithm to detect anti-symmetric pairs just as easily as normal symmetric pairs. This principle extends to anti-symmetric pairs of all six types.

Anti-symmetry exists with respect to any permutation group, but only the anti-symmetries with respect to variable pairs appear to be useful.

As with partial, total and multi-phase symmetry, anti-symmetries can be detected using variable pair relations when no library exists.

### Kronecker symmetry

**Fig. 21:**
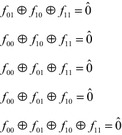
The Kronecker symmetries.

As with multi-phase and anti-symmetries, the Kronecker symmetries (Chrzanowska-Jeske 1999) of a Boolean function $f$ are defined with respect to two input variables $x_{i}$ and $x_{j}$. Let $f_{00}$, $f_{01}$, $f_{10}$, and $f_{11}$ be the cofactors of $f$ with respect to these two variables. There are five types of Kronecker symmetry, defined by the five relations given in Fig. 21. In this figure, $\hat{0}$ represents the constant zero function.

**Fig. 22:**
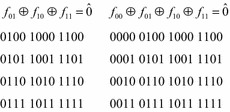
Boolean Orbits for Kronecker symmetries.

If the function under test a 4-input function with input variables $x_{1}$, $x_{2}$, $x_{3}$, and $x_{4}$. The Boolean orbits of the Kronecker symmetries are given in Fig. 22.

As with other Boolean orbits, each orbit is reduced to a characteristic function. Because the vectors of each orbit are not required to be mapped to the same value, one cannot use the implication relation to detect Kronecker symmetries. Instead i compute the function $D_{i} = f\& C_{i}$ for each orbit $O_{i}$, and count the number of input vectors that are mapped to 1 by $D_{i}$. (The result cannot exceed the number of vectors in the orbit.) If the result is an even number for each orbit, then $f$ possesses the associated Kronecker symmetry. Note that the function $D_{i}$ can be used to compute the implication relation, since $C_{i}$ implies $f$ if and only if $D_{i} = C_{i}$, and implies $\overline{f}$ if and only if $D_{i} = \hat{0}$.

**Fig. 23:**
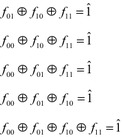
The negative Kronecker symmetries.

There are also symmetries known as the *Negative* Kronecker symmetries, defined by the relations of Fig. 23, where $\hat{1}$ represents the constant-one function.

One proceeds exactly as before, but in this case, after obtaining $D_{i}$, and counting the vectors mapped to 1, one checks for an odd number instead of an even number.

The USD algorithm can detect Kronecker symmetries using variable-pair relations when no libraries exist.

### Matrix-based symmetry

As pointed out in (Maurer 2011), it is possible to characterize the symmetry of an *n*-input Boolean function in terms of $$ n \times n $$ permutation matrices instead of degree-*n* permutations. When this is done, the matrices are assumed to be over the field $$ GF(2), $$ which has two elements, zero and one, and which uses the AND and XOR functions in place of multiplication and addition. The advantage of doing this is that any group of $$ n \times n $$ non-singular matrices can be used as a symmetry group, and there are many more matrix groups than there are permutation groups. The number of degree-*n* permutations is $$ n! $$, while the number of $$ n \times n $$ non-singular matrices is given by the following formula.

$$ G(n) = \prod\limits_{i = 0}^{n - 1} {\left( {2^{n} - 2^{i} } \right)} $$

The number $G(n)$ grows much faster than $n!$. [For example, 6! = 720, but G(6) is over 20 million.] Because of the large number of non-singular matrices, the opportunities to detect symmetry are much greater. There are many types of matrix-based symmetry that have no counterparts in permutation-based symmetry. Conjugate symmetry (Maurer 2011) is just one of these.

**Fig. 24:**
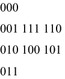
A matrix group and its Boolean orbits.

Just like permutation-based symmetry, matrix-based symmetry can be characterized in terms of Boolean orbits. The Boolean orbits of a matrix group can be computed using the same algorithm as for permutation groups. Figure 24 shows a matrix group that has no counterpart in permutations, and gives the Boolean orbits for it.

Like permutation groups, the symmetry generated by a matrix group is completely determined by its Boolean orbits. Theorem 5 applies to matrix groups as well as to permutation groups, so the same principle can be used to detect symmetry with respect to matrix groups. Of course, the set of all matrix groups is much too large to permit a library of all possible subgroups to be constructed. It is therefore necessary to focus on known symmetry types such as conjugate symmetry and the subgroups of these symmetries. For such symmetry types, it is possible to generate the library entries on the fly using the standard libraries as a basis.

### Other types of symmetry

**Fig. 25:**
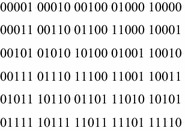
Boolean orbits for $R_{5}$.

The USD algorithm has dynamic generators for rotational and dihedral symmetry. The symmetry groups in question are $R_{n}$ for rotational symmetry of degree *n*, and $D_{2n}$ for dihedral symmetry of degree *n*. The output from these generators is normally used on the fly and discarded, but it could easily be placed in a library for future use. To create a library entry for rotational symmetry one starts with an n-element vector and rotates it n − 1 times to create each orbit. This normally creates orbits of size n, but sometimes they are smaller. For example, the vector 101010 yields an orbit of size 2. Figure 25 shows the orbits for rotational symmetry of degree 5.

For *n* < 6 rotational and dihedral symmetry are the same. Dihedral symmetry includes all *n* rotations of a vector, but also includes the mirror image of a vector. (The mirror image of $(v_{1} ,v_{2} ,v_{3} ,v_{4} )$ is $(v_{4} ,v_{3} ,v_{2} ,v_{1} )$.) For *n* < 6, the mirror image of a vector can be obtained by rotating the vector. However, for *n* = 6 and larger, there are vectors whose mirror image cannot be obtained in this fashion. For 6 inputs, the vector 001011 has a mirror image of 110100, but no rotation of 001011 will produce 110100. This means that for *n* = 6 and larger, that dihedral symmetry and rotational symmetry are distinct.

To create an entry for dihedral symmetry of degree *n*, one first starts with rotating each vector $n - 1$ times, and then one reverses each vector to produce the required orbits.

Another type of symmetry, that is used to simplify Boolean functions, is Auto Symmetry. Auto symmetry occurs when an *n*-input Boolean function becomes an $n - k{\text{-input}}$ function under a linear transformation of its inputs (Bernasconi et al. 2008).

**Fig. 26:**
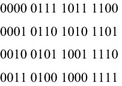
Autosymmetry Boolean orbits.

As explained in (Bernasconi et al. 2008) autosymmetric functions take constant values on a subspace of $\{ 0,1\}^{n}$ and its affine spaces. The subspace and its affine spaces constitute the Boolean orbits of the autosymmetry. Autosymmetry is detected in the same way as ordinary symmetry. Figure 26 gives an example of a set of autosymmetry orbits. Autosymmetry orbits are always all the same size. Every element of $\{ 0,1\}^{n}$ appears in one of the orbits, and the size of the orbits is a power of 2.

Although hierarchical symmetry has been extensively studied (Kravets and Sakallah 2000, 2002), the USD algorithm not treat it as a distinct type of symmetry. As with other symmetries, hierarchical symmetries are specified as groups of permutations. In group theory, a hierarchical symmetry is defined by a permutation group that is the *wreath product* (Robinson 1995) of two or more other permutation groups. The existing libraries contain many examples of wreath product groups.

### Experimental data

Many experiments were run to test the efficacy of the USD algorithm. All experiments were on modest hardware: a Dell laptop containing an Intel P9500 Core 2 Duo 2.53 Ghz CPU with 3.48 GB of RAM and Windows XP Professional with Service Pack 3. Initially, the experiments focused on analyzing all functions with 5 or fewer inputs primarily to gauge the speed of the algorithm. Functions with 2 and 3 inputs are trivial, because a 2-input function is either non-symmetric or totally symmetric. A 3-input function is non-symmetric, totally-symmetric, or partially symmetric in two variables.

Four-input functions are more interesting. In addition to total and partial symmetries, there is dihedral symmetry and lock-step symmetry between two variable pairs. The term “lock-step symmetry” describes non-independent sub-symmetries between two or more, not necessarily disjoint, sets of variables. The only example of this for 4-input functions is exemplified by the function $x_{1}^{\prime } x_{2} + x_{3}^{\prime } x_{4}$ from “Background”. The symmetry group for this function is {I, (1, 3)(2, 4)}, where the two transpositions (1, 3) and (2, 4) must operate in lock-step with one another. Strictly speaking, lock-step symmetry is not a type of symmetry, but a catch-all term to describe a phenomenon that occurs in many different ways. The term *set*-*symmetry* (Mohnke et al. 2002) has been used to describe symmetries such as that defined by the group {I, (1, 3)(2, 4)}, but similar phenomena can occur in many other ways. Describing the ways in which “lock-step” symmetries arise would require an extensive discussion of advanced group theory, and would add little or nothing to the understanding of the USD algorithm.

**Fig. 27:**
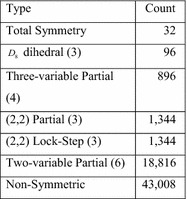
All 4-input functions.

There are 65,536 4-input functions and 4,294,967,296 5-input functions. The results of the analysis of 4-input functions took 2 s of real time and are given in Fig. 27. This figure combines results for the symmetries that are conjugate to one another. The number of conjugates is given in parentheses following the symmetry type. Each function is counted only once. There are subgroups of $S_{4}$, such as K4 and $A_{4}$, which are not listed, because they are forbidden.

**Fig. 28:**
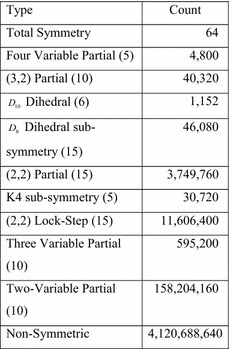
All 5-input functions.

As Fig. 27 shows, the proportion of symmetric functions to non-symmetric functions decreases as the number of inputs increases. The results of the analysis for all 5-input functions took about 4 h of real time and are given in Fig. 28.

It is not possible to obtain a complete analysis of all *n*-input functions, where *n* > 6. Even for *n* = 6, such an analysis would require many of thousands of years on existing hardware because the total number of functions is 18,446,744,073,709,551,616. This is unfortunate, because the number of different types of symmetry explode for six or more inputs. For five and fewer inputs, the types of symmetry are more-or-less what one would expect. For six and more inputs, the variety is astounding, and some types are less than intuitive and quite difficult to describe. For example, $S_{6}$ contains a class of subgroups which is isomorphic to the *wreath product* of $S_{2}$ and $S_{3}$, a class that is the *split extension* of $S_{3} \times S_{3}$ by $S_{2}$, and another that is the *non*-*split extension* of $S_{2}$ by $S_{3}$. In $S_{8}$ there is a class that is isomorphic to the *quaternion group*. (These are only a few examples out of many.) One needs considerable expertise in group theory even to understand what these groups are. Describing their effects is quite difficult. When the number of inputs is greater than seven, even classification by the techniques of advanced group theory fails. In (Pfeiffer 2005) there are several subgroups of $S_{7} - S_{12}$ that are classified as “a group of size *n*,” with no further explanation.

**Fig. 29:**
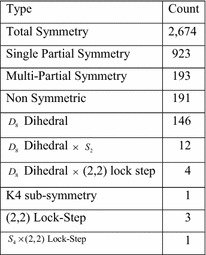
The ISCAS85 benchmarks.

Figure 28 reinforces the idea that as the number of variables increases, the proportional number of non-symmetric functions decreases. However, this does not necessarily describe what happens with functions that are used in practice. To determine what sort of functions one would encounter in practice, experiments were conducted with the ISCAS85, LGSynth89 and LGSynth91 benchmarks. The initial results for ISCAS85 were uninformative because the standard description of these circuits is in terms of individual gates whose symmetries are obvious. To obtain more substantive results the gates of the ISCAS85 circuits were combined into larger groups and each group was treated as a single function. The method for doing this was to identify the fanout-free regions of each circuit, and treat each such region as a function. (A fanout-free circuit has a single output, and can easily be treated as a single function.) Because there are no complete libraries for circuits with more than eight inputs, the fanout-free regions were partitioned into subcircuits containing no more than eight inputs. This procedure was expected to produce mostly partial and total symmetries, and this was indeed the case, but there were also some interesting surprises. Figure 29 summarizes the results for total and partial symmetry and gives the counts of the interesting cases.

**Fig. 30:**
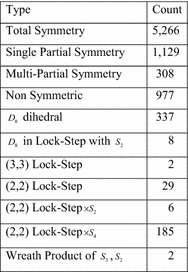
The LGSynth89, and LGSynth91 benchmarks.

To further study the symmetries of (more or less) real circuits, tests were run on the LGSynth89 and LGSynth91 benchmarks. The results of these tests are given in Fig. 30. Figures 29 and 30 clearly show that the circuits used in practice contain far more symmetries than randomly chosen circuits. This emphasizes the importance of correctly detecting the symmetries of a function.

The USD is implemented as a collection of objects representing, functions, orbits, symmetry rules (a group and its orbits), rule libraries and function libraries. The low-level implementations were hidden from the user, to enable many different implementations to be used without affecting the high-level algorithms. The only requirement for the USD algorithm to function correctly is the ability to compute the implication relation. For compressed libraries and sub-symmetries it is also necessary to compute cofactors and the product of a permutation with a function.

The current implementation models functions as compressed truth tables, which is an array of 64-bit integers with one bit for each input vector. This implementation is extremely efficient for up to six inputs, but rapidly becomes less efficient as the number of inputs increases beyond this point. A single 64-bit integer will suffice for up to six inputs. Beyond six inputs, the number of integers doubles for each input, with 16–20 inputs being the practical limitation. For more general circuits, Binary Decision Diagrams or something similar would almost certainly more efficient.

**Fig. 31:**
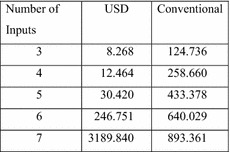
Seconds per 1,000,000 functions.

To further gauge the efficiency of the algorithm, the amount of real time required to determine the symmetry of 1,000,000 functions was measured. The results are reported in Fig. 31. For three and four input functions, it was necessary to test the same functions repeatedly. For five, six and seven inputs, the first 1,000,000 functions were tested. It should be noted that the efficiency of the algorithm depends heavily on the underlying implementation, so these numbers should be taken only as rough guidelines.

The superior speed of the USD algorithm is obvious for 3–6 inputs. The superior speed is also evident for the 7-input test when one remembers that the conventional algorithm is testing for 877 different symmetries while the USD algorithm is testing for 11,300 different symmetries. (Twelve times as much work for three and a half times as much time.)

The categorization of all 4-input functions was virtually instantaneous, as were the experiments with the ISCAS85, LGSynth89 and LGSynth91 benchmarks. The categorization of all 5-input functions took about 4 h.

For eight or more inputs, the conventional approach will normally be faster than the USD algorithm. However the results will be considerably less satisfactory, since only a small fraction of the available symmetries will be detected.

The reader is cautioned that these results are valid only for the current implementation of the USD algorithm. It is extremely likely that much more efficient implementations will be developed in the future, necessitating a new characterization of the algorithm’s performance.

## Conclusions

The USD algorithm is a powerful tool that can be used in many different contexts. It is a simple, yet powerful and efficient algorithm for detecting virtually any type of symmetry. It is my belief that many types of symmetry *could* be exploited if there were methods to detect them. Because the USD algorithm makes these types accessible, it is expected that significantly more exploitation of symmetry will take place in the future.

The initial paper on detecting symmetry in Boolean functions was published in 1949 (Shannon 1949), and since that time the basic principle introduced in this paper has been refined and improved many times. I believe that the USD algorithm is similar to (Shannon 1949) in that it is a beginning rather than an end. I hope that the principles elucidated here will be refined and improved in the same way that Shannon’s principle has been refined and improved over the years.

One area of potential improvement is in the parallelization of the USD algorithm. The USD algorithm does many comparisons, virtually all of which are independent of one another. Library searches could easily be parallelized, because searching one entry is only loosely connected to searching other entries. While it is true that the USD algorithm searches the largest subgroups first, these searches tend to be faster than the searches for smaller subgroups because there are fewer Boolean orbits. This would allow the faster parallel searches to abort the slower ones once a match is found. Searching an individual library entry could also be easily parallelized since the testing of one Boolean orbit does not depend on the outcome of any other test. When large numbers of functions are being tested, each function can be tested in parallel with the others. It is probable that that the USD algorithm could be parallelized in such a way as to take advantage of virtually any available parallelism.

As for my own plans, the future development of the USD algorithm will include the incorporation of at least a portion of the $S_{9}$ through $S_{18}$ material, as well as the identification and incorporation of new parameterized symmetry types. In particular, I am interested in creating automatic generators for various types of matrix-based symmetry.

In any case, the USD algorithm should prove to be a powerful tool that can be used in many different areas of Electrical Design Automation.
